# An efficient outlier removal method for scattered point cloud data

**DOI:** 10.1371/journal.pone.0201280

**Published:** 2018-08-02

**Authors:** Xiaojuan Ning, Fan Li, Ge Tian, Yinghui Wang

**Affiliations:** Department of computer science and Engineering, Xi’an university of technology, Xi’an, Shaanxi, China; Universidad de Valladolid, SPAIN

## Abstract

Outlier removal is a fundamental data processing task to ensure the quality of scanned point cloud data (PCD), which is becoming increasing important in industrial applications and reverse engineering. Acquired scanned PCD is usually noisy, sparse and temporarily incoherent. Thus the processing of scanned data is typically an ill-posed problem. In the paper, we present a simple and effective method based on two geometrical characteristics constraints to trim the noisy points. One of the geometrical characteristics is the local density information and another is the deviation from the local fitting plane. The local density based method provides a preprocessing step, which could remove those sparse outlier and isolated outlier. The non-isolated outlier removal in this paper depends on a local projection method, which placing those points onto objects. There is no doubt that the deviation of any point from the local fitting plane should be a criterion to reduce the noisy points. The experimental results demonstrate the ability to remove the noisy point from various man-made objects consisting of complex outlier.

## Introduction

**Fig 1:**
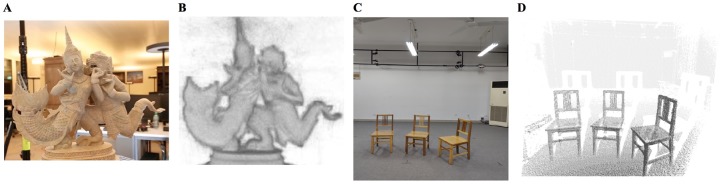
Two examples of laser scanning scene. A and B are respectively the artwork image and its point cloud in [6]. C and D display the indoor scene image and its ccorresponding point cloud data.

Scanning object with complex geometry and varying surface reflectiveness, the collected scanned point cloud may contain extensive outliers, which are inevitable by-products of 3D scanning [1–3]. As illustrated in Fig 1, we can see that it is prone to producing outliers and noise in the PCD due to occlusion or sensor imperfections. The resulting point clouds are thus often noisy, and this inevitably destroys fine details. Outlier points, usually unorganized, noisy, sparse, and inconsistent in local point density, have geometrical discontinuities, arbitrary surface shape with sharp features [4]. Sparse and dense outliers pose much more problematic issues to the applications of the scanned point cloud, especially in 3D shape analysis [5], object modeling [6] and object recognition [7]. Therefore, how to remove outliers from scattered point cloud data is the main focus of this paper.

Compared to the common manual removal process, which is time consuming and relies on the operator’s experience, it is highly desirable to develop an automatic outlier removal method. However, automatic and effective removal of outliers is challenging since the scanned object is unavailable and the estimation of the object surface shape would be inaccurate in the presence of extensive outliers.

In this paper, the outliers are classified into three categories: sparse outlier, isolated outlier and non-isolated outlier. We proposed an automatic method to remove those outliers based on local density and local projection. The sparse outlier can be detected easily according to low local point density. Although isolated outlier often formed clusters because of high point density, the local density may have a lower value when the local area is large enough. The two types of noise both can be detected and removed by the method based on local density. However, the non-isolated outlier is close to the model, we proposed to project those outliers locally onto the original object through the local fitting plane. Different from other denoising methods, the procedure does not remove noisy point but project noisy point onto the local fitting plane to make the model more regular. We can obtain the noise-free model through the two methods and prove that our method is effective to denoise the point cloud model.

## Related work

Scanned PCD acquired is usually polluted by noise for the existence of the scanner system’s inherent error and aircraft’s shock. In this research, the purpose of outlier removal is to identify and remove outliers efficiently in scanned PCD. Outliers can be removed by applying a spatial depth-pass filter to the 3D point data [8]. Many scholars home and abroad do research on this problem that is sorted in two kinds: discontinuous operators-based method and surface fitting-based method.

### Discontinuous operators-based method

Wang et al. [9] utilized a distance-based deviation factor to detect sparse outlier and then detected small outlier clusters using region growing. Rusu et al. [10] proposed an efficient approach to detect sparse outlier, which correspond to low point densities. In practice, however, the local density of scanned PCD for good surface points can be non-uniform and incomplete. Chenot [11] proposed a new method to aberrant outliers on a wider range of blind separation instances. Based on sparse signal modeling, it makes profit of an alternate reweighting minimization technique that yields a robust estimation of the sources and the mixing matrix simultaneously with the removal of the spurious outliers. Shao et al. [12] reconstructed dense depth maps from sparse point clouds and used them to remove points that are in significant visibility conflict and to augment the input point cloud. Similarly, a free space constraint was used to clean up depth maps in [13]. Unfortunately, non-isolated outlier clusters were not considered. Also methods based on robust descriptor and wavelet transform are also effective to reduce the noisy points. Zhang et al [14] proposed a distance-based method to detect the outliers. Tola et al. [15] used a robust descriptor for large-scale multi-view stereo matching in order to reduce the amount of outliers in the computed point cloud. The parameterization-free projection operator [16] results in a resampled point cloud by means of point projections, but onto a multivariate median, being more robust to noise and able to detect outliers. By taking into account the point density, the method was extended to deal with sharp features [17] and a high level of non-uniformity [18].

### Surface fitting-based method

Carsten et al. [19] presented a new method for anisotropic fairing of a point sampled surface using an anisotropic geometric mean curvature flow. Desbrun et al. [20] developed methods to rapidly remove rough features from irregularly triangulated data intended to portray a smooth surface. The main task is to remove undesirable noise and uneven edges while retaining desirable geometric features. Zeng et al. [21] combined the Moving Least Square surface fitting with Lagrange operator to implement point cloud filtering. Zheng et al. [22] proposed a point cloud filtering method based on variable radius circle and B-spline fitting, the filtering precision of the algorithm is improved 1 to 5 times of the traditional methods, it can be used for the city, mountains and forest. Weyrich et al. proposed three novel methods to detect outliers including the plane fitting criterion, mini-ball criterion and nearest-neighbor reciprocity criterion [23]. Shao et al [24] presents a novel outlier removal method which is capable of fitting ellipse in real-time under high outlier rate.

All methods mentioned above have the advantage of implementation friendly. The outlier removal methods based on discontinuous operators aforementioned generally focus on a certain type of outliers and are inapplicable to other types of outliers. It is more robust in sparse outlier detection and removing small clusters of outliers. But points on the edges also have a high proportion of unidirectional neighbors and will be detected. Non-isolated outliers are usually ignored. Although method based on surface fitting can deal with non-isolated outliers, it is too complicated and too time-consuming to be applied, and it also requires the continuity of data.

In the paper, we give two algorithms to response various types of outliers. The method based on local density have handled on isolated outlier cluster and sparse outlier and the method based on local projection can well trim non-isolated outliers. At the same time, small maintenance overhead is inevitable when we adopt the two methods.

## Overview

**Fig 2:**
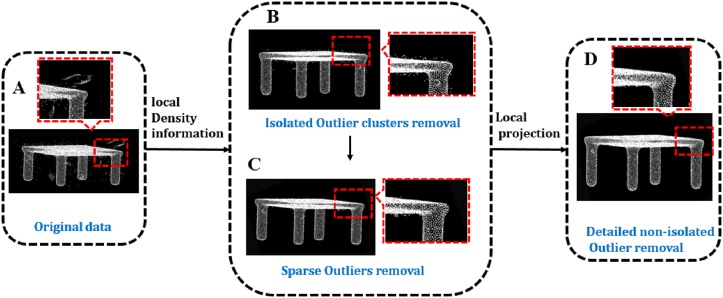
Overview of our method.

The input to our method is the raw scan of 3D object and real scene, represented as unorganized point clouds. Generally, the scanning data collected from reality are often noisy, uncertainty and incomplete. Lots of denoising methods exits, yet less can successfully deal with all type of noisy point. In order to achieve this goal, we propose a novel outlier removal method on the basis of two visual characteristics. Analyzing different kinds of noisy points, we raise different methods to solve the corresponding issue. Fig 2 displays the overview of our proposed algorithm, highlighting our method and the processing steps. Our algorithm essentially consists of local density based and local projection based method.

Our denosing algorithm removes outliers from a set of input point clouds {**p_i_**|*i* = 1, 2, …, *N*} by analyzing different category of outliers and their distribution.

Outlier Distribution Analysis. A method considering the outlier distribution and the distance distribution is proposed to detect those outliers that could be easily found in object.

Isolated outlier clusters and sparse outlier removal. Local density based method was proposed to detect and remove the isolated outlier clusters and sparse outlier.

Detailed non-isolated outlier removal. In order to remove the remaining outliers from the detected shapes, a criterion is provided by the deviation of any point from the plane that fitted by its neighborhoods (we called it local fitting plane). According to the deviation information we project the noisy points to local fitting plane to trim the model.

For the original data with various outliers in Fig 2(A), the method based on local density information is used to remove isolated outlier clusters (in Fig 2(B)) and sparse outlier (in Fig 2(C)). After the two steps, the data would be further trimmed (in Fig 2(D)) by the local projection based method.

## Sparse outlier and isolated outliers removal

### Outlier distribution analysis

**Fig 3:**
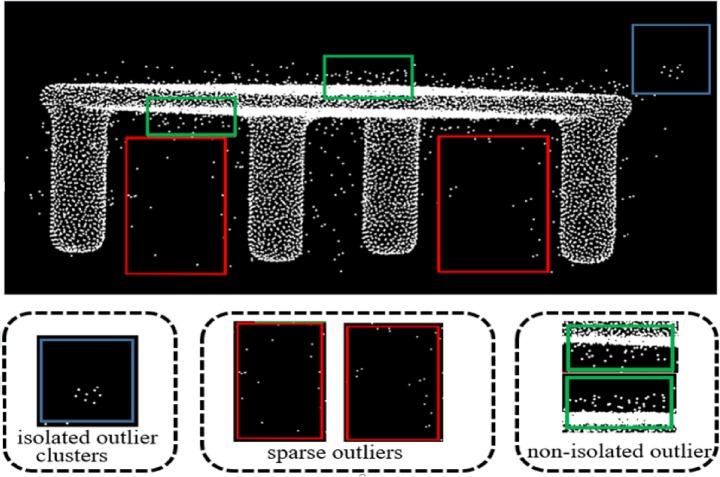
Different outlier distribution.

In this work, our outlier removal method is developed to effectively identify sparse outlier, isolated outlier clusters, and non-isolated outlier clusters in scanned point clouds, demonstrated in Fig 3.

Sparse outlier are erroneous measurement points with low local point density.

Isolated outlier have high local point density and are relatively separated from the scanned data.

Non-isolated outlier are attached to the scanned surface and cannot be easily separated.

In Fig 3, the outliers marked by red box belong to sparse outlier whose point density is smaller than others. Also the density of the isolated outlier clusters is remarked by blue box. So the two kinds of noisy point will be removed according to the density information. The non-isolated outlier in green box is near to the model. We will not remove these noisy points but project them to the local fitting plane. Using different strategies to deal with different kinds of noisy points we can achieve final denoising.

### Local density analysis

In this section, we detail the density estimation applied to remove the isolated outlier clusters and sparse outlier. The scattered point cloud denoted as **P** = {**p_1_**, **p_2_**, **p_3_**, …, **p_N_**}, finding the optimal neighborhood of each point is important for computing the local covariance matrix of each point. The distribution of mobile laser point clouds has variable point densities because of occlusion, varying scanning angles, and varying distances to the laser scanner. Let the *k* Nearest Neighbor points of **p_i_** be *KNN*(**p_i_**), i.e. **Q** = {**q_1_**, **q_2_**, **q_3_**, …, **q_k_**}. Our algorithm removes inconsistent points from point cloud **P** by analyzing their geometric information and density.

To determine the density information, each point originating from scanning data has to be examined over the *k* nearest neighborhood. The local density is obtained by calculating the average distance of **p_i_** to its *k* nearest neighborhood **q_j_**(**j** = **1**, …, **k**). The average distance of **p_i_** is defined as

d¯i=1/1kk·∑j=1kdist(pi,qj) (1)

where *i* = 1, 2, …, *k* and **dist**(**p_i_**, **q_j_**) is the Euclidean distance between **p_i_** and **q_j_**. The local density function **LD**(**p_i_**) of **p_i_** is defined as Eq 2:

$$\begin{array}{c} \text{LD}(p_{i})=\frac{1}{k}\sum_{q_{j}\in \text{KNN}\left(p_{i}\right)}\exp(\frac{-\text{dist}(p_{i},q_{j})}{{\bar{d}}_{i}}) \end{array}$$ (2)

where *k* is the number of nearest neighborhood, ${\bar{d}}_{i}$ is the average distance between **p_i_** and **q_j_**. The probability of point belongs to outlier can be defined as Eq 3:

$$\begin{array}{c} pro\left(p_{i}\right)=1-\text{LD}\left(p_{i}\right) \end{array}$$ (3)

*pro*(**p_i_**) ∈ [**0**, **1**]. The greater the value of *pro*(**p_i_**), the more likely it is to be outliers.

Then we will decide whether the point **p_i_** would be kept based on the local density *pro*(**p_i_**). We retain the point **p_i_** if it satisfies the following condition that

$$\begin{array}{c} pro(p_{i})<\delta \end{array}$$ (4)

During the test, an appearance was found that the threshold *δ* was not fixed for all models. *δ* is different for different models because different models have complex settings in the scanning process. In practice, we choose *δ* as a fixed ration of $\bar{d_{i}}$, i.e. $\delta =0.1\cdot \bar{d_{i}}$.

The pseudo code of our sparse outlier and isolated outlier removal algorithm is defined in Algorithm 1. Setting proper threshold *δ* for the probability of point belongs to outlier and removing those points beyond the threshold *δ*, the model can discard the isolated outlier cluster and sparse outlier.

**Fig 4:**
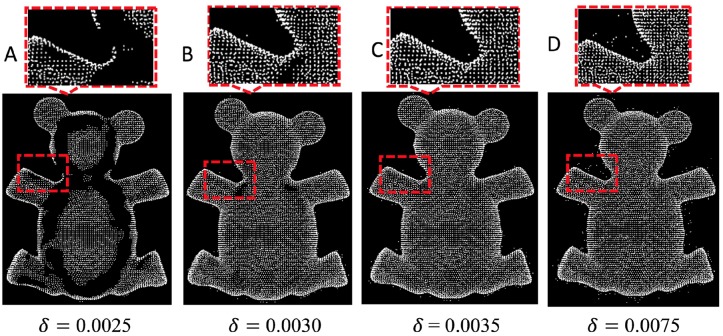
Different *δ* in our method.

Fig 4 illustrate the effect of *δ* on a simulation study. We show a denoised point cloud with different *δ*. When *δ* = 0.0025, most of the points in bear model are deleted. When *δ* = 0.0075, the outlier removal method does not work.

**Algorithm 1** Non-isolated outlier removal algorithm

1: **Input**: Three dimensional scanned PCD with various outliers

2: **Output**: sparse outlier and isolated outlier removal results

3: **for** all point **p_i_** in **P** do

4:  search the *k* nearest neighborhood of **p_i_**, i.e. *KNN*(**p_i_**)

5:   calculate the local density **LD**(**p_i_**) of **p_i_**

6:    compute the probability *pro*(**p_i_**)

7: **end for**

8: sort the *pro*(**p_i_**) in ascending order

9: compare the first N number of *pro*(**p_i_**)

10: **for** *i* = 1: *N*

11:  **if** *pro*(**p_i_**) > *δ*

12:  **then** delete **p_i_** as outliers

13: **end for**

## Non-isolated outlier removal

The non-isolated outlier is very close to the surface of object, it is difficult to remove from the object since it may often cause errors or even remove the original point in object. We proposed a method that converting these outliers to object surface points. In this section, we will illustrate how to project those non-isolated outlier onto their corresponding fitted plane. Fitting local plane is the crux of the method based on local projection in local neighborhood, which we called it local fitting plane.

### Local fitting plane

A plane is parameterized by its normal vector **n** = {**n_x_**, **n_y_**, **n_z_**} and any given point on the plane. In order to obtain the local fitting plane, we first compute its normal vector.

Generally, the principle components analysis (PCA) is performed by computing the eigenvalues and eigenvectors to evaluate the normal vector of plane. A minimal ellipsoid is obtained by enclosing the *k* nearest neighborhood of a point **p_i_**. Let $\bar{p}$ be the centroid and *M* be the 3 × 3 covariance matrix defined as Eq 5:

$$\begin{array}{c} M=\frac{1}{k}\sum_{i=1}^{k}\left(p_{i}-\bar{p}\right){\left(p_{i}-\bar{p}\right)}^{T} \end{array}$$ (5)

where **p_i_** ∈ **P**, λ_0_, λ_1_ and λ_2_ are the eigenvalues of *M* and λ_0_ ≤ λ_1_ ≤ λ_2_. In essence, λ_0_, λ_1_ and λ_2_ respectively represent the length of the three semi-principal axes of the ellipsoid in 3D. The eigenvector of the smallest eigenvalue is the approximation of normal vector at the vertex **p_i_**. $\bar{p}$ is the center point of *k* nearest neighborhood of point **p_i_**. The local fitting plane **L_i_** can be represented accordingly.

### Detailed non-isolated outlier removal

**Fig 5:**
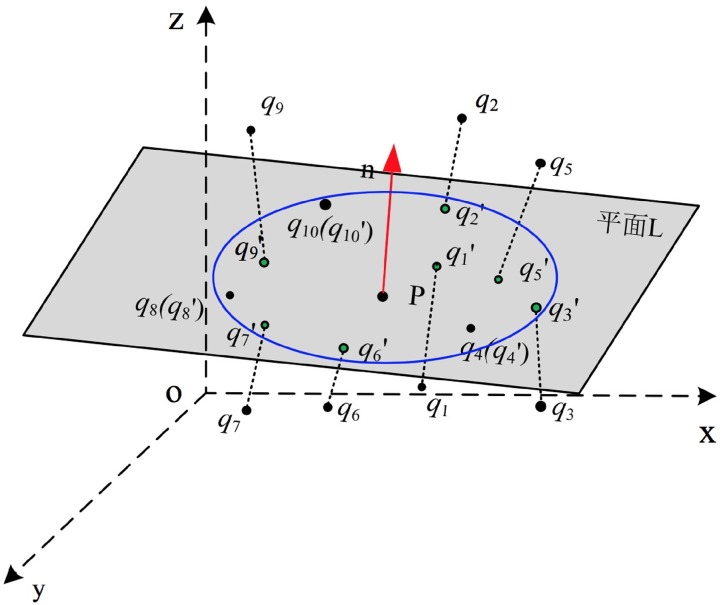
Local fitting plane and projection of point.

In this section, the target is to project those outlier points onto the local plane **L_i_**. Fig 5 displays the local fitting plane **L_i_** of point **p_i_** and the process of putting forward the neighboring point **q_j_** to the local plane.

The normal vector **n** = {**n_x_**, **n_y_**, **n_z_**}, ∥**n**∥ = 1 and a given point **p_0_** can parameterize the plane **L_i_**. The deviation of **q_j_** from the plane **L_i_** is given by Eq 6:

$$\begin{array}{c} dis_{p_{i}q_{j}}=\left(q_{j}-p_{0}\right)\cdot n \end{array}$$ (6)

Then projection of point **q_j_** onto the corresponding fitted plane is to push **q_j_** along the opposite direction of normal vector **n**. The projected **q**_**j**_′ is defines as Eq 7:

$$\begin{array}{c} {q_{j}}^{\prime }=\left(q_{j}-{\text{dis}}_{p_{i}q_{j}}\cdot n\right) \end{array}$$ (7)

**Algorithm 2** Non-isolated outlier removal algorithm

1: **Input**: Three dimensional scanned point cloud data with various outliers

2: **Output**: non-isolated outlier removal

3: **for** all point **p_i_**(**i** = **1**, **2**, …, **N**) in **P** do

4:  search the *k* nearest neighborhood **q_j_**(**j** = **1**, **2**, …, **k**)

5:  fitting a local plane **L_i_** for **p_i_** and **q_j_**

6:  compute the deviation $dis_{p_{i}q_{j}}$ of **p_i_**, **q_j_** from plane **L_i_**

7:  project **q_j_** onto the corresponding fitted plane **L_i_**

8:  the projected point **q_j_**′ is the new coordinate of **q_j_**

9: **end for**

**Fig 6:**
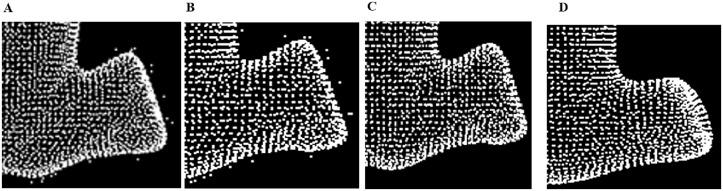
The selection value of *k*. A: pending data. B: *k* = 4. C: *k* = 10. D: *k* = 50.

The key step is to fix the local fitting plane and the rationality of the fitting plane depends on the value of *k* (*k* Nearest Neighbor). A bigger *k* may cause deformation of the model and a smaller *k* may offer an invalid fitting plane and an invalid projection. Fig 6 shows the results with different *k*.

## Experimental results

**Fig 7:**
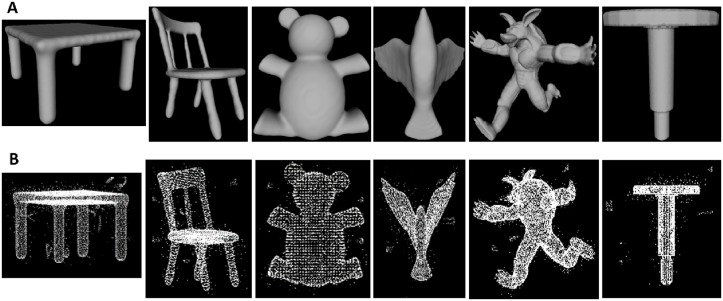
3D models from Princeton shape Database. A: Original model. B: point cloud model with outliers.

We experimentally evaluate our method primarily using scanned point cloud contain various categories of noise. The datasets used in our experiments include:

1. A gallery of models in Fig 7 is chosen from Princeton shape Database and variety of Gaussian noise is added in the models.
2. Several real indoor scene data are also selected.
3. Datasets from [6] are also selected, including DRAGON, TORCH, and STATUE models.

We first describe the experimental setting of our method and then demonstrate the results using our method. Meanwhile we summarize the performance of our algorithm where accuracy and completeness errors as well as runtime.

Experimental setting. Our method is implemented using C++ and run on a desktop PC with an Intel I7-6700 CPU (quad core, 3.4 GHz) and AMD Radeon R5 340X graphics card.

### Results on 3D models

**Fig 8:**
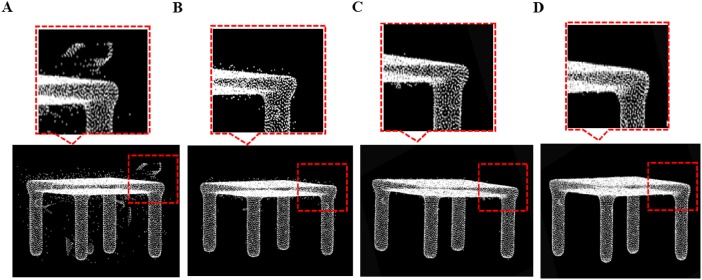
Outlier removal for table model. A: Table model with different outliers, B: isolated outlier removal, C: sparse outlier are removal, D: non-isolated outlier removal result.

We run our algorithm to six 3D models: chair, table, bird, monster, bear, and Nail. We demonstrate the experimental results of our method for those models in Fig 7. Fig 8(A) is table model which contains all kinds of outliers, after the local density based processing, isolated outlier is deleted greatly in Fig 8(B) and 8(C). The final data in Fig 8(D) having been trimmed after using local projection based method. It can show that our method is very efficient for denoising of point cloud data.

**Fig 9:**
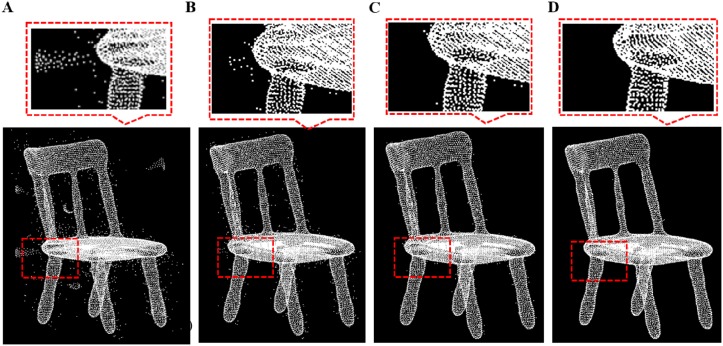
Outlier removal for chair model. A: Chair model with different outliers, B: isolated outlier removal, C: sparse outlier removal, D: non-isolated outlier removal result.

**Fig 10:**
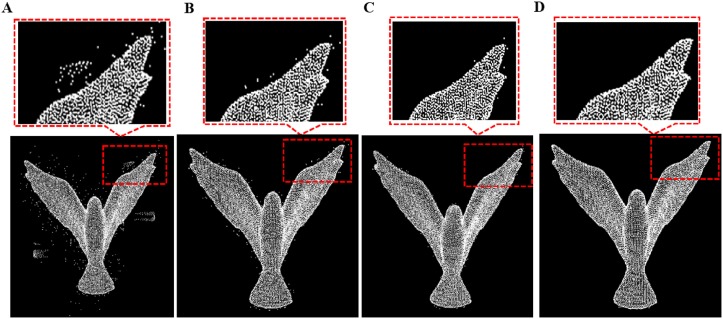
Outlier removal for bird model. A: Bird model with different outliers, B: isolated outlier removal, C: sparse outlier removal, D: non-isolated outlier removal result.

**Fig 11:**
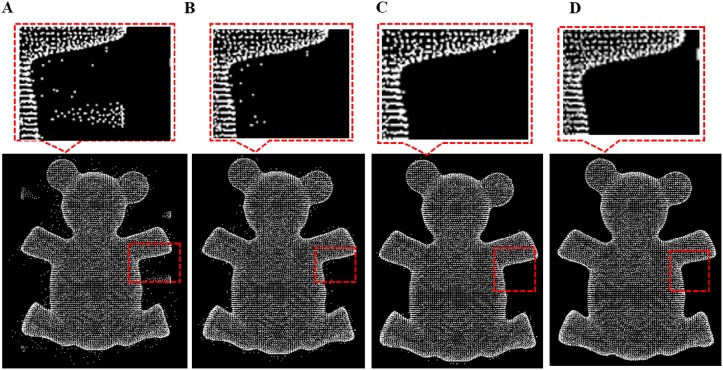
Outlier removal for bear model. A: Bear model with different outliers, B: isolated outlier is removed, C: sparse outlier is removed, D: non-isolated outlier removal result.

**Fig 12:**
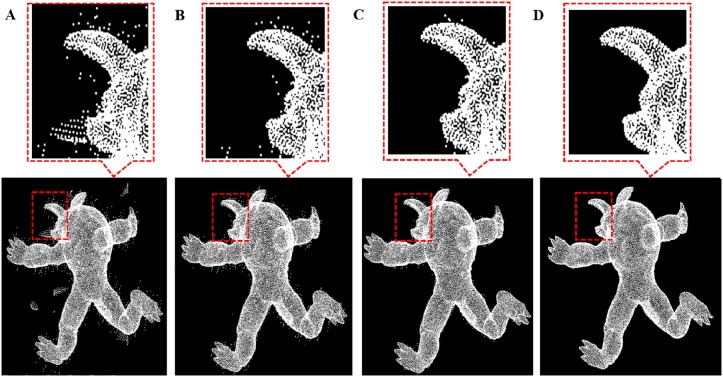
Outlier removal for monster model. A: Monster model with different outliers, B: isolated outlier is removed, C: sparse outlier is removed, D: non-isolated outlier removal result.

**Fig 13:**
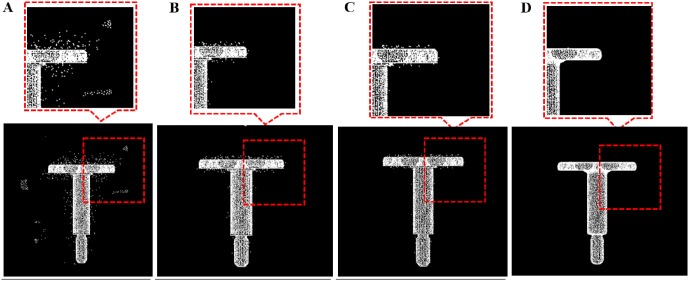
Outlier removal for Nail model. A: Nail model with different outliers, B: isolated outlier is removed, C: sparse outlier is removed, D: non-isolated outlier removal result.

**Fig 14:**
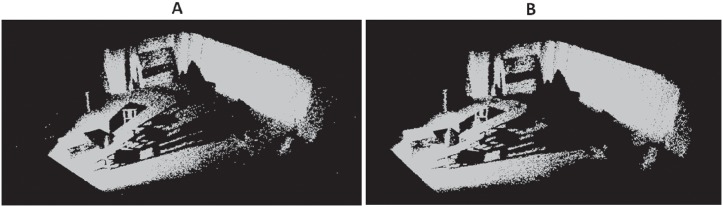
Outlier removal for indoor scene S1. A: Original Indoor scene S1, B: Outlier removal result.

**Fig 15:**
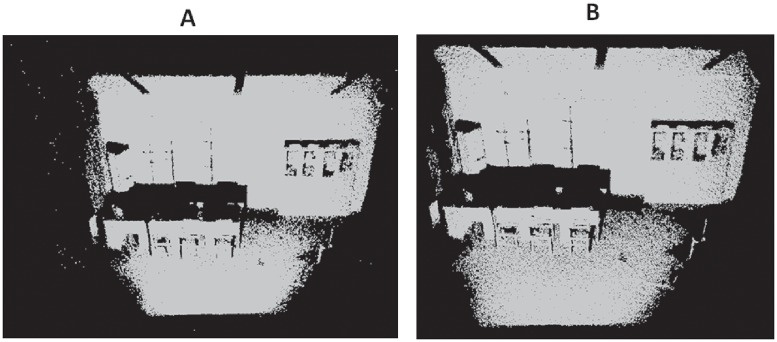
Outlier removal for indoor scene S2. A: Original Indoor scene S2, B: Outlier removal result.

Figs 9–13 demonstrate respectively the experimental results for chair, bird, monster and bear model. In addition, to test the robustness of our method we run our method to two scanned real-life indoor scenes with 146044 points and 153218 points respectively. These real-life scenes contain a variety of object clutters and more outliers. Figs 14 and 15 demonstrate the two original scenes and the outlier removal results.

### Comparison against other denoiser

**Fig 16:**
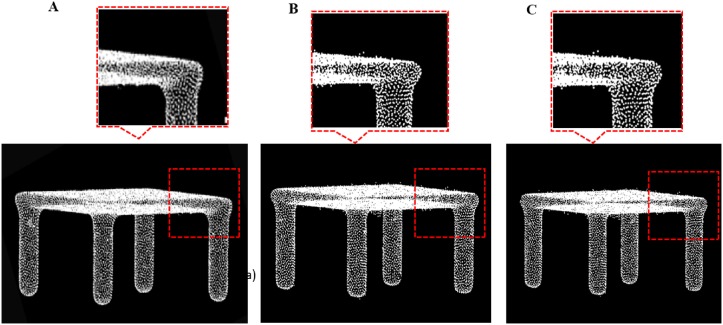
Comparison result for table data between our outlier removal method and the classic two method. A: Our methods, B: Statistics-based method; C: Radius based method.

**Fig 17:**
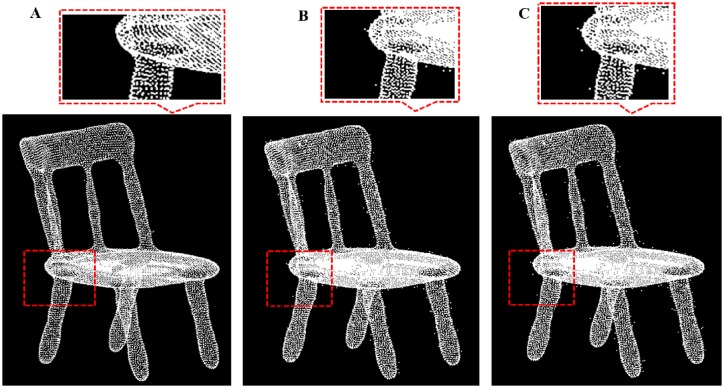
Comparison result for chair data between our outlier removal method and the classic two method. A: Our methods, B: Statistics-based method; C: Radius based method.

**Fig 18:**
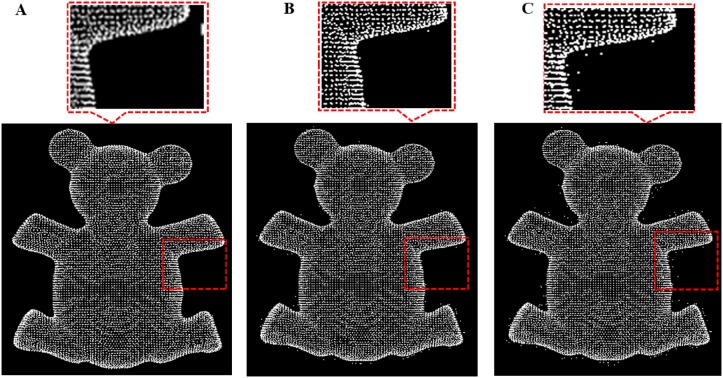
Comparison result for bear data between our outlier removal method and the classic two method. A: Our methods, B: Statistics-based method; C: Radius based method.

**Fig 19:**
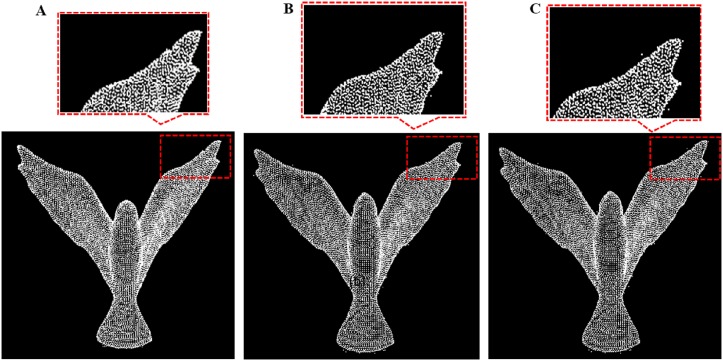
Comparison result for bird data between our outlier removal method and the classic two method. A: Our methods, B: Statistics-based method; C: Radius based method.

**Fig 20:**
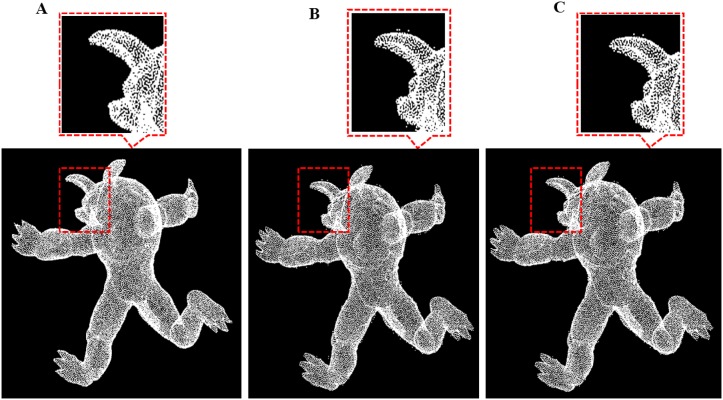
Comparison result for monster data between our outlier removal method and the classic two method. A: Our methods, B: Statistics-based method; C: Radius based method.

**Fig 21:**
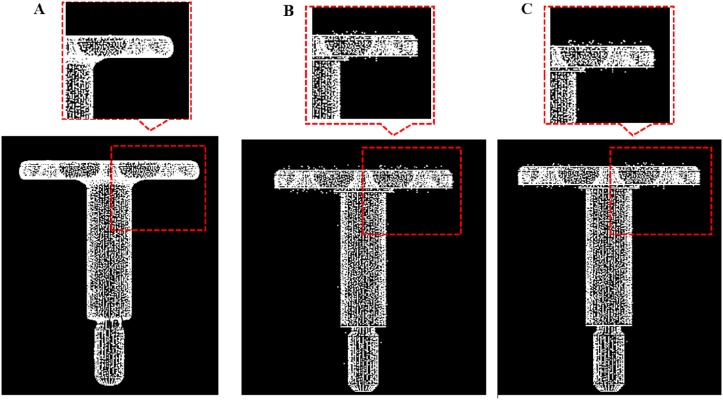
Comparison result for Nail data between our outlier removal method and the classic two method. A: Our methods, B: Statistics-based method; C: Radius based method.

**Fig 22:**
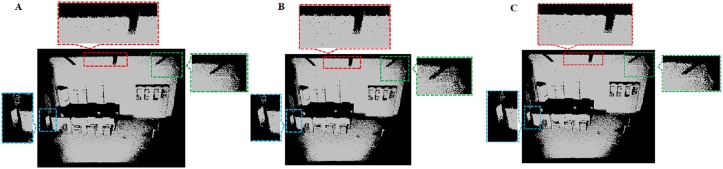
Comparison result for indoor scene S1 between our outlier removal method and the classic two method. A: Statistics-based method; B: Radius based method; C: Our methods.

**Fig 23:**
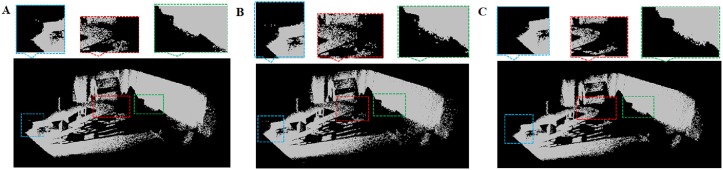
Comparison result for indoor scene S2 between our outlier removal method and the classic two method. A: Statistics-based method; B: Radius based method; C: Our methods.

We compare our outlier removal method with two alternative methods (radius-based method and statistics-based method) in Point Cloud Library respectively. We evaluate the quality of our outlier removal system by running it on the six test models and test scenes S1, S2, and output the final point clouds after removing different outliers in Figs 16–23. As shown in these figures, when there are sparse outlier and isolated outlier, both methods could obtain reasonably good removal results. When the non-isolated outliers exist, however, the quality of results decreases significantly. In contrast, our method produces good result even for indoor cluttered scenes.

**Fig 24:**
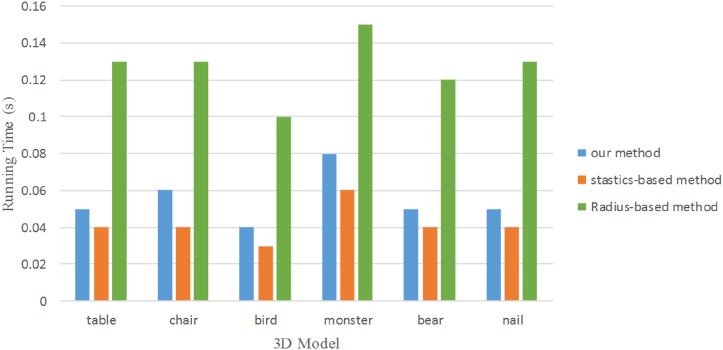
Running time comparison result for six 3D models.

**Fig 25:**
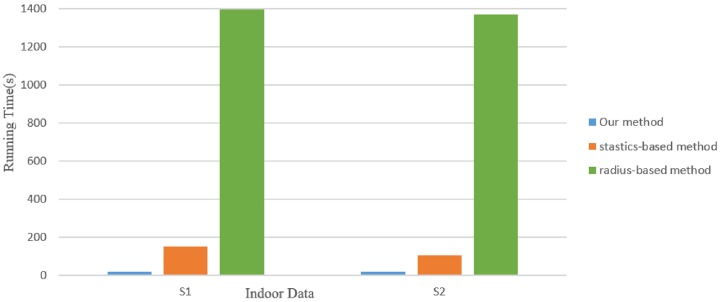
Running time comparison result for two indoor scene data.

Fig 24 plots the running time of our method for all the 3D models in the paper. For comparison, we also plot the results of a radius-based method and statistics-based method. The statistics-based method takes less time than ours, but the quality of outlier removal result is weak. As shown in Fig 25, it plots the running time of our method for two indoor scenes S1 and S2. We can see that our method takes less time than the other two methods when the point number of object increase significantly. Our outlier removal method is robust, making it particularly effective for removing different kinds of outliers.

**Fig 26:**
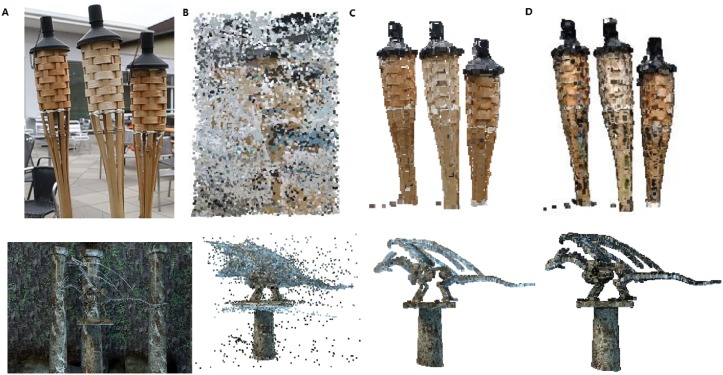
Comparison results on TORCH and DRAGON models. A: Datasets, B: Point cloud; C: Our outlier removing method. D: Wolff et al. [6].

**Fig 27:**
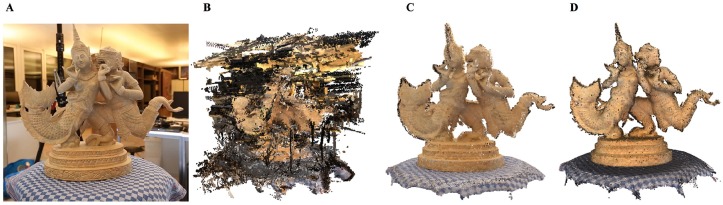
Comparison results on STATUE model. A: Datasets, B: Point cloud; C: Our outlier removing method. D: Wolff et al. [6].

**Fig 28:**
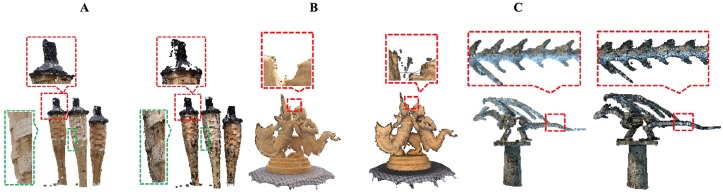
Detail comparison results. A: TORCH model, B: DRAGON model; C: STATUE model. (left: our method, right: method in [6]).

In Figs 26–28, we compare our method with other point cloud denonising method in [6]. We can see that the fine detailed information (such as smooth boundary) are retained for DRAGON model in Fig 28(C). For the TORCH model, our method can remove the noise without missing any point in data, however the method in [6] would lead to data missing.

### Performance analysis

We summarized the performance of our algorithm, where accuracy and completeness errors as well as the running time were measured. Table 1 reports timing and some related statistics of our algorithm running over different models. The value are, from left to right, the model, the number of original data *N*, the number after removing the isolated outlier clusters *N*_−_ *i*, the number after removing the sparse outlier is *N*_−_ *s*, the total time that method takes *t* and the ratio of detected noise $P_{c}=1-\frac{N_{-}s}{N}$.

**Table 1:** Timings and statistical data of point model by our method.

model	*N*	*N*_−_ *i*	*N*_−_ *s*	*t*	*P*_*c*_
table	15845	15433	14968	0.05	0.055
chair	17623	17113	16565	0.06	0.060
bird	13180	12742	12470	0.04	0.054
monster	28246	27510	26588	0.08	0.059
bear	15365	14931	14461	0.05	0.059
Nail	15009	14655	14507	0.05	0.033

To access the results more quantitatively, we measured the bias of the reconstructed meshes from ground truth results. We evaluate the accuracy and completeness of each mesh according to the metrics used in [25]. We measured errors in terms of accuracy (in world units) and completeness (in percent), using an accuracy threshold of 90%, and a completeness threshold of 0.005 world units. Table 2 shows the accuracy and completness of 3D models after using our methods. Tables 3 and 4 illustrates the timings and parameter setting in our method.

**Table 2:** Performance analysis of our algorithm on 3D models.

model	Accuracy	Completeness
table	0.004197	93.4%
chair	0.00303	100%
bird	0.004293	93.6%
monster	0.002769	97.3%
bear	0.002205	100%
Nail	0.005783	87.3%

**Table 3:** Timings and parameter setting of 3D model.

Model	Original Data Number	After Removal	Time(s)	*k*	*δ*
TORCH	3604726	2946940	2421.391	50	0.05
DRAGON	5601652	4815690	514.392	400	0.1
STATUE	5849643	4767875	789.507	100	0.0005

**Table 4:** Parameter setting in our method.

Model	*k* (sparse outlier and isolated outlier removal)	*δ*_*i*_	*k* (non-isolated outlier removal)
table	80	0.005	10
chair	75	0.004	10
bird	70	0.003	10
monster	50	0.0015	10
bear	50	0.0035	10
nail	50	0.0015	10

We give the complexity of two key algorithmic components. The complexity is *O*(*N*ß*logN*) for the local density based method, with *N* being the number of points in an object, and *O*(*NlogN*) for the local projection based method.

### Limitation

As our method rely on the points distribution and need to calculate the local density of points, our method might fail for very dense outliers exist. When there are denser outliers, it may lead to deformation of object after removing the outliers. To eliminate this problem, we plan to adopt learning-based strategy that could estimate and distinguish the outlier from original points.

## Conclusion

In this paper, a robust method is presented in this paper to effectively remove isolated outlier, sparse outlier, and non-isolated outlier from scanned objects in point cloud. The local density and the deviation from the local fitting plane provide a fundamental way. The local density based method can remove sparse outlier and isolated outlier. The deviation of point from the local fitting plane should be a criterion to reduce the non-isolated outlier. Experimental results demonstrate the ability to remove complex outliers from various man-made objects. As demonstrated, the presented method is able to achieve robust results in removing three types of outliers and preserving distinct geometric features such as sharp edges in a scanned point cloud.

## Supporting information

S1 Data Indoor scene S1. (OBJ)

S2 Data Indoor scene S2. (OBJ)
